# NSAIDs and Cardiovascular Diseases: Role of Reactive Oxygen Species

**DOI:** 10.1155/2015/536962

**Published:** 2015-09-20

**Authors:** Rajeshwary Ghosh, Azra Alajbegovic, Aldrin V. Gomes

**Affiliations:** ^1^Department of Neurobiology, Physiology, and Behavior, University of California, Davis, CA 95616, USA; ^2^Department of Physiology and Membrane Biology, University of California, Davis, CA 95616, USA

## Abstract

Nonsteroidal anti-inflammatory drugs (NSAIDs) are the most commonly used drugs worldwide. NSAIDs are used for a variety of conditions including pain, rheumatoid arthritis, and musculoskeletal disorders. The beneficial effects of NSAIDs in reducing or relieving pain are well established, and other benefits such as reducing inflammation and anticancer effects are also documented. The undesirable side effects of NSAIDs include ulcers, internal bleeding, kidney failure, and increased risk of heart attack and stroke. Some of these side effects may be due to the oxidative stress induced by NSAIDs in different tissues. NSAIDs have been shown to induce reactive oxygen species (ROS) in different cell types including cardiac and cardiovascular related cells. Increases in ROS result in increased levels of oxidized proteins which alters key intracellular signaling pathways. One of these key pathways is apoptosis which causes cell death when significantly activated. This review discusses the relationship between NSAIDs and cardiovascular diseases (CVD) and the role of NSAID-induced ROS in CVD.

## 1. Introduction

Nonsteroidal anti-inflammatory drugs (NSAIDs) are the most widely used over-the-counter drugs as well as the most prescribed class of drugs for a variety of conditions including pains, rheumatoid arthritis, osteoarthritis, musculoskeletal disorders, and other comorbid conditions [1]. Millions of people suffer from pain resulting in the prolonged use of NSAIDs being common. Besides reducing or relieving pain NSAIDs have been shown to be useful as anticancer agents in various kinds of cancers [2–4]. However, NSAIDs also have undesirable side effects including ulcers [5], bleeding [6], kidney failure [7, 8], and increased risk of heart attack and stroke [8, 9]. One of the mechanisms which has been associated with the adverse effects of NSAIDs is the generation of oxidative stress. The present review focuses on NSAIDs-induced ROS generation leading to cardiovascular diseases (CVD).

## 2. Types of NSAIDs

NSAIDs may be classified according to their mechanism of action. Nonselective NSAIDs like ibuprofen and naproxen, which comprise one class, inhibit both cyclooxygenase-1 (COX-1) and cyclooxygenase-2 (COX-2) enzymes. A second class of NSAIDs (celecoxib and rofecoxib) targets only the COX-2 pathway and is termed as COX-2 selective inhibitors (also known as coxibs). COX selectivity is one of the determining factors that is considered when administrating NSAIDs to a patient. Administration of nonselective NSAIDs has been associated with side effects like peptic ulcer disease and gastrointestinal bleeding [10]. COX-2 selective NSAIDs have been shown to exhibit gastroprotective effects unlike the nonselective NSAIDs and are thus useful in patients with painful gastrointestinal conditions [10–12]. Another class of semiselective NSAIDs (indomethacin, meloxicam, and diclofenac) have a higher affinity for COX-2 but tend to inhibit the COX-1 pathway also [13]. However, irrespective of their mechanism of action, prolonged exposure to any class of NSAIDs has been shown to have potential adverse effects on cardiovascular events in patients with or without preexisting cardiovascular conditions, depending on the duration and dosage of these drugs [14, 15] (Table 1). Patients with preexisting cardiovascular conditions such as coronary artery disease, hypertension, and history of stroke are at the greatest risk of cardiovascular events after taking NSAIDs [14, 15]. Patients who have recently had cardiovascular bypass surgery are advised not to take NSAIDs due to a high risk of heart attacks [16, 17]. The increased selectivity for COX-2 has also been reported to increase the risk of various CVD [18, 19]. Meta-analyses of several trials have shown that coxibs are associated with a high risk of atherothrombotic vascular events [20].

## 3. Mechanism of Action of NSAIDs

**Figure 1:**
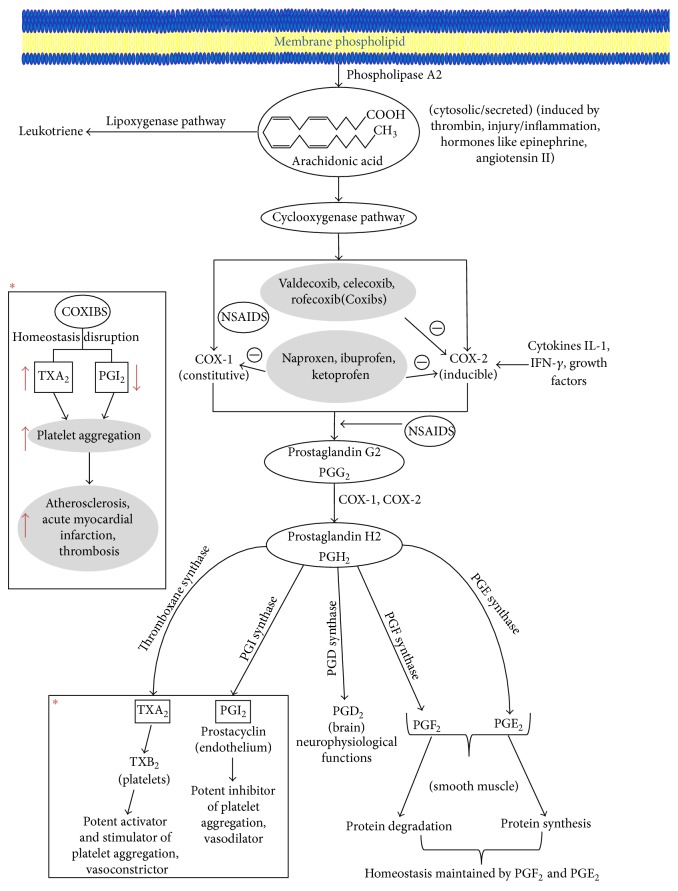
Inhibition of cyclooxygenase pathway by NSAIDs. Coxibs as well as nonselective NSAIDs inhibit the formation of the metabolites of the cyclooxygenase pathway thereby disrupting the homeostasis maintained by these metabolites. Coxibs cause an imbalance between the levels of thromboxane and prostacyclin being more favorable towards thromboxane and decreasing prostacyclin levels leading to the aggregation of platelets and causing thrombosis.

NSAIDs exert their pain relieving effect mainly by inhibiting the cyclooxygenase pathway (Figure 1). This pathway is responsible for the conversion of arachidonic acid to prostaglandins and thromboxanes [21]. Although COX is officially known as prostaglandin-endoperoxide synthase (PTGS) the abbreviation “COX” is commonly used for cyclooxygenase-1 and cyclooxygenase-2 in medicine. In genetics, the abbreviation PTGS is officially used for the cyclooxygenase family of genes and proteins to prevent ambiguity with the cytochrome c oxidase family of genes and proteins which are also abbreviated COX. Arachidonic acid is the main precursor to the formation of various eicosanoids in the cyclooxygenase pathway. Of all the metabolites formed in the arachidonic acid metabolism, thromboxane A2 in the platelets is the major product which along with prostacyclin (prostaglandin I2) maintains vascular homeostasis [22] (Figure 1). Both these eicosanoids (thromboxane A2 and prostaglandin I2) have opposing effects. While thromboxane is well known for its role in vasoconstriction and aggregation of platelets, prostacyclin is important for platelet aggregation inhibition and vasodilation.

The COX enzyme is present as two isoforms, each with distinct functions: COX-1 is constitutively expressed in the stomach, kidneys, intestinal mucosa, and other tissues [23]. It protects the mucosal lining of the stomach and plays an important role in vasoconstriction and platelet aggregation [23]. On the other hand the inducible COX-2 is upregulated during times of inflammation where it causes vasodilation [24]. COX-1 and COX-2 are similar in molecular weights, 70 and 72 kDa, respectively, and show 65% homology with near-identical catalytic sites (based upon information from UniProtKB/Swiss-Prot database). The critical difference between the isoenzymes, which permits the selective inhibition of each isoform, is the substitution of isoleucine 523 in COX-1 with valine in COX-2 [25]. The presence of valine, which is a smaller amino acid than isoleucine, allows drugs entrance to a hydrophobic side-pocket only accessible in COX-2. Expression of both isoforms, COX-1 and COX-2, may be upregulated and downregulated under various pathological conditions [26]. It is likely that the classification of the COX enzymes into two isoforms was an oversimplification [26] as COX-2 may be constitutively expressed in the brain [27], kidney [28], and testes [29]. In fact immunohistochemical studies have revealed the constitutive expression of COX-2 mRNA in the lung, thyroid gland, spleen, and adipose tissue, which was greater than COX-1 in these tissues, and in the liver both isoforms were expressed equally [29]. Therefore the idea that COX-2 can only be expressed under inducible conditions is unlikely since recent evidence suggests their occurrence in various human tissues under normal conditions.

Coxibs disrupt the balance between the levels of thromboxane A2 and prostaglandin I2 leading to atherosclerosis, thrombosis, and other cardiovascular complications. Coxibs, through their selective inhibition of COX-2, inhibit endothelial cell synthesis of prostacyclin [30]. In the ApoE−/− mice (model for atherosclerosis), deletion of the prostacyclin receptor increased atherogenesis with no such effect observed in thromboxane receptor deleted mice [30]. Apart from its role in the inhibition of cyclooxygenase pathway, NSAIDs have been shown to cause cell death by the inhibition of the Akt signaling pathway [31], downregulation of the NF-*κ*B pathway [32], downregulation of the Bcl pathway [33], upregulation of the nonsteroidal activated gene-1 [34], and altering the p53 pathway [35], all of which have been suggested to be involved in apoptosis [36]. Apoptosis (programmed cell death) induced by NSAIDs has been suggested to be due to oxidative stress caused by increased generation of reactive oxygen species (ROS) [37].

**Figure 2:**
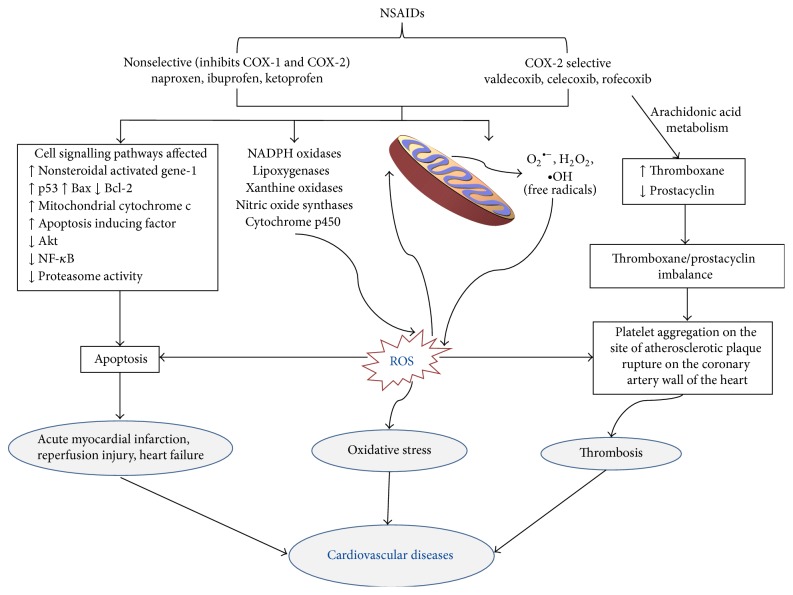
Pathways involved in the development of cardiovascular diseases by NSAIDS. The figure shows the upregulation and downregulation of various pathways by NSAIDs leading to the development of CVD. Mitochondria play a major role in the generation of ROS induced by NSAIDs followed by oxidative stress and finally CVD.

A series of mechanisms are involved wherein NSAIDs exert their cardiotoxic effects and cause various cardiac conditions. Various non-NSAID drugs like doxorubicin, azidothymidine, and cisplatin have been shown to induce oxidative stress as a consequence of elevated ROS levels [38]. Doxorubicin, for example, induced cardiotoxicity through DNA damage and apoptosis in cardiac cells as a result of oxidative stress which were reduced by the antioxidant effect of statin [37]. It is possible that the oxidative stress induced by NSAIDs, which is known to cause apoptosis and cell death, is significantly involved in causing cardiovascular dysfunction (Figure 2).

## 4. Incidences of CVD Induced by NSAIDs

Various clinical trials have been made during the past few years regarding the safety and effectiveness of NSAIDs in CVD (Table 2). Most trials focused on the CVD outcome of NSAID use in patients with a previous history of cardiovascular disease. Few trials were carried out on patients with no history of CVD. An important finding is that not only nonselective NSAIDs lead to the development of hypertension in both normotensive and hypertensive individuals [39], but their use interferes with the antihypertensive medications except for the calcium channel blockers [40]. The risk of atrial fibrillation, heart failure, myocardial infarction, and other cardiovascular conditions also increased in patients with a history of these pathological conditions (Table 2).

The CVD related outcomes in patients enrolled in the REACH (REduction of Atherothrombosis for Continued Health) registry showed that in patients with established stable atherothrombosis, use of NSAIDs increased the incidences of myocardial infarction and cerebrovascular conditions [41]. Additionally, this paper reported that the use of NSAIDs with other antiplatelet drugs (except aspirin) increased the rate of cardiovascular events like cardiovascular death, myocardial infarction, and stroke [41]. Although a few studies suggest that COX selectivity does not seem to be a determining factor for myocardial infarction [13, 42], several studies suggest that coxibs elevate the rate of incidences of CVD compared to nonselective NSAIDs [18–20]. Several clinical trials have been completed and are still ongoing, but some inconsistencies in the results exist regarding the effect of different types of NSAIDs on cardiovascular outcomes (Table 2). One meta-analysis report found that ~75% studies investigating the cardiovascular risk in new NSAID users reported an increase in the occurrence of cardiovascular diseases within the first month of NSAID use [43]. Overall, the different trials showed that several NSAIDs increased the risk of CVD both at low and high doses [41, 43–45]. Targeted inhibition of COX-2, even for a short term, was found to increase the risk of atherothrombosis [46]. The COX-2 selective NSAID rofecoxib at low doses (50 mg daily) increased the occurrences of myocardial infarction by 0.5% in approximately 8000 patients with rheumatoid arthritis compared to 0.1% in those treated with naproxen (500 mg twice daily), indicating a severe thrombogenic effect of rofecoxib compared to naproxen [47]. The main increased risk of cardiovascular events associated with COX-2 inhibitors was an increased risk of myocardial infarction [46].

The report of Adenomatous Polyp Prevention on Vioxx (APPROVe) trial on the adverse effect of rofecoxib on CVD ultimately led to the discontinuation of this drug in several countries [48]. Increased thromboembolic events have been associated with rofecoxib compared to naproxen [49]. In another trial rofecoxib was found to be associated with cardiac arrhythmias and renal conditions [50]. However similar adverse effects were not encountered in patients treated with other COX-2 inhibitors [50]. More importantly, several semiselective NSAIDs like diclofenac and meloxicam and nonselective NSAIDs including naproxen and ibuprofen have also been shown to increase the incidences of CVD [46, 51]. Of these drugs, diclofenac has been shown to increase the occurrences of myocardial infarction and stroke even at lower doses of <150 mg/day compared to naproxen at doses of >1000 mg/day [51]. This effect of diclofenac has been attributed to its greater selectivity for COX-2 inhibition. It has also been reported that ibuprofen is associated with CVD comparable to the effect of COX-2 selective inhibitor, celecoxib [20]. It has been suggested that the difference between the effect exhibited by rofecoxib and other NSAIDs of the same class on cardiovascular incidences is due to the distinct chemical properties and prooxidant activity of rofecoxib [52]. The toxic effect of rofecoxib was reportedly due to its ability to reduce the low density lipoprotein (LDL) antioxidant capacity as a result of increased lipid peroxidation [52]. Merck voluntarily withdrew rofecoxib (Vioxx) in 2004. Pfizer was asked by the US Food and Drug Administration (FDA) to withdraw valdecoxib (Bextra) from the market in 2005 because of a higher than expected number of reports of serious and potentially life-threatening skin reactions and deaths.

Naproxen seems to show less cardiovascular events than other commonly used NSAIDs, possibly because it mimics the activity of acetylsalicylic acid (aspirin) by suppressing cyclooxygenase platelet thromboxane B2 [53]. However, the role of naproxen in the development of CVD has been controversial. Several studies have reported the cardioprotective effect of the compound [54–56], and on the other hand increased cardiovascular risk has been associated with the use of this NSAID [57–59]. The only NSAID which has not been associated with increased cardiovascular events is aspirin. Aspirin is the only known NSAID which has antithrombotic activity through the inhibition of platelet aggregation in the artery of the heart thereby exhibiting cardioprotective effects [60]. It does so by acetylating the platelet COX-1 and irreversibly suppressing thromboxane A2 production, which is required for platelet aggregation and thrombus formation [60]. Naproxen on the other hand causes reversible inhibition of cyclooxygenase by binding to the enzyme [61]. It was found that inhibition in thromboxane A2 synthesis was more pronounced (75%) compared to prostacyclin inhibition (50%) after the oral administration of 500 mg naproxen in healthy volunteers [61].

## 5. NSAIDs and Reactive Oxygen Species Generation

NSAIDs have been shown to be associated with increased ROS production (Table 3). In the heart the main producer of ROS is the mitochondria [62]. Under normal physiological conditions, mitochondria generate ROS as a consequence of aerobic respiration [63]. During aerobic respiration about 5% of O_2_ consumed via aerobic reaction is converted into ROS [63]. The mitochondria-dependent overproduction of ROS has been reported under numerous pathological conditions including myocardial heart failure, inflammatory diseases, cancer, hypertension, and diabetes [64–67]. In myocardial heart failure, cardiomyocytes have been shown to be targeted by excessive ROS generation [64].

ROS levels and the redox state of a cell are considered to be important in the dysfunction of various biological signaling pathways. The formation of ROS via the reduction of molecular oxygen or by the oxidation of water leads to the formation of free radicals such as superoxide anion (O_2_^•−^), hydroxyl radical (^•^OH), and hydrogen peroxide (H_2_O_2_). Oxidative stress arises when the oxidant production (sum of all the ROS) surpasses the antioxidant capacity in the cells. Under normal physiological conditions low amounts of O_2_^•−^ in the cardiomyocytes are converted to the less toxic H_2_O_2_ by the enzyme superoxide dismutase (SOD). The reaction further proceeds by the formation of water by the action of the enzyme catalase or glutathione peroxidase (GPx) system [68]. However, when a homeostatic imbalance between the cellular antioxidant capacity and ROS levels occurs, elevated ROS levels can damage cellular macromolecules including lipids, proteins, and nucleic acids. High levels of ROS also accelerate cell death due to apoptosis as well as necrosis by the activation of poly(adenosine diphosphate ribose) polymerase [69] and thus significantly contribute to the development of various pathological conditions [70].

The generation of ATP in the mitochondria utilizes the electrons from reduced substrates that are transferred to an acceptor molecule of the electron transport chain (ETC). Leakage of electrons from the mitochondria ETC results in O_2_^•−^ formation [71]. Electron leakage is potentially possible at 9 sites in the ETC; however most of the ROS seem to be associated with complexes I and III which have been well studied [72]. The generation of ROS increases in intact mitochondria as well as in submitochondrial particles due to the oxidation of complex I substrates as a result of inhibiting complex III by antimycin A [72]. On the other hand, rotenone, an inhibitor of complex I, prevented the antimycin A induced ROS generation in mitochondria but not in the submitochondrial particles [72].

The ROS status in the cellular system regulates many biological processes. While increased levels of ROS have been shown to be involved in various pathological conditions, under basal conditions, the generation of free radicals in the heart is needed for cellular responses including regulating myocyte growth and maintaining vascular smooth muscle tone [73]. Basal levels of ROS play an important role in the increase of cell cycle progression and intracellular signaling associated with phosphorylation of several signaling proteins like mitogen-activated protein kinases (MAPKs) and protein kinase B [74]. Under normal physiological conditions, ROS upregulates the Akt signaling pathway and promotes cell survival [75]. ROS also behaves as second messengers in signaling pathways wherein it has been demonstrated that, in the presence of basal ROS levels, tyrosine phosphatase activity is higher relative to its kinases [76]. Ligand stimulation (as in the case of neutrophils, through the binding of cytosolic proteins to a membrane bound oxidase) leads to increased ROS levels and deactivation of tyrosine phosphatases with a consequent increase in the kinase activity [76]. This condition is transient and is reversed by reductions in ROS levels. ROS has also been demonstrated to be involved in the modulation of transcription factors like NF-*κ*B, Hif-1 [77], and several cardiac transcription factors like SRF, Sp1, AP-1, GATA-4, and MEF2C [78].

Current experimental data suggest that the main mechanism through which NSAIDs exert their anticancer activity is through the generation of ROS leading to oxidative stress and finally apoptosis in cancer cells [79–82]. ROS is accompanied by the activation and inhibition of several signaling pathways associated with cell death and cell survival although controversies exist regarding the role of ROS in the downregulation and upregulation of these pathways [83, 84]. The Akt pathway is one of the most important pathways for promoting cell survival and growth, and it has been shown that high ROS levels are lethal and can inactivate the Akt signaling pathway [85]. NF-*κ*B is an important transcription factor involved in cell survival, inflammation, and stress responses. Its downregulation was demonstrated by exposure of HLEC (human lymphatic endothelial cells) cells to sustained oxidative stress [86]. Increased levels of H_2_O_2_ caused by addition of glucose oxidase to HLECs prevented NF-*κ*B(p65) regulated gene expression by blocking translocation of NF-*κ*B to the nucleus [86]. NF-*κ*B regulated gene expression is important for a broad range of physiological processes [86].

Sulindac, a nonselective NSAID, as well as its metabolites, generates ROS in different cancer cell lines [80–82]. Similarly, diclofenac-induced apoptosis of various kinds of cancer cells has been reported [87]. This apoptosis is mainly mediated by an increase in the intracellular levels of the ROS [87]. In the cardiovascular system the major sources of ROS generation include the mitochondria, NADPH oxidases, xanthine oxidoreductases, lipoxygenase, cyclooxygenases, nitric oxide synthases, and cytochrome P450 based enzymes (Figure 2). Continuous exposure of cardiovascular cells to oxidative stress associated with elevated ROS levels would result in altered cellular homeostasis which could be an important contributing factor for various cardiovascular conditions. Endothelial cells play a critical role in maintaining vascular homeostasis which is important for the control of many cardiovascular diseases including atherosclerosis and thrombosis [88]. In human umbilical vein endothelial cells (HUVEC), 160 *μ*M sulindac induced endothelial apoptosis as determined by a time dependent increase in annexin V positive cells. Both sulindac and indomethacin significantly increased cleaved poly(ADP-ribose) polymerase (PARP) levels as well as increasing the level of the apoptotic activating factor caspase-3 [89]. The apoptosis in HUVEC cells induced by the NSAIDs was associated with reduced PPAR*δ* and 14-3-3-*ε* expression. Under normal conditions 14-3-3-*ε* binds to phosphorylated Bad inhibiting translocation of Bad to the mitochondria and preventing apoptosis through the mitochondrial pathway. However, sulindac through the suppression of 14-3-3-*ε* expression increased Bad translocation to mitochondria thereby inducing apoptosis [89].

## 6. Mitochondria Are the Main Target Organelles of the NSAIDs

It has been shown that the heart is more susceptible to ROS generation induced by drugs like doxorubicin compared to other tissues of the body although the drugs are evenly distributed throughout the body [90]. This is possibly due to high levels of mitochondria in the heart which are the major producers of ROS in the cardiovascular system. Over 90% of ATP required for the normal functioning of the heart is provided by the mitochondria, which utilizes an efficient oxidative phosphorylation system. ATP production may increase depending upon the requirements of the body, especially at times of excessive physical exertion or other hormonal stimulations [91]. Mitochondria are not only the major producers of the free radicals [72], but excessive generation of ROS in turn targets the mitochondria itself [92]. NSAIDs have been shown to have adverse effects on the mitochondria resulting in the increased production of ROS [93, 94]. In yeast cells different NSAIDs generated ROS which was associated with delayed growth in wild-type cells [95]. Yeast cells lacking mitochondrial DNA were resistant to a delay in cell growth [95]. More specifically the yeast deletion strains lacking the genes encoding subunits of the mitochondrial complexes III and IV were significantly resistant to diclofenac as well as indomethacin and ketoprofen [95]. This data suggests that mitochondria are the main target organelles through which these compounds exert their toxic effect on these cells. The data also suggest that mitochondrial complex III and/or complex IV are affected by NSAIDs resulting in increased ROS production.

The nonselective NSAID indomethacin has been shown to target mitochondria by directly inhibiting mitochondrial complex 1 of human colonic adenocarcinoma cells. The inhibition of complex I was accompanied by a decrease in the intracellular ATP levels within 10–30 minutes [96]. Studies directly related to mitochondrial dysfunction or damage in the presence of NSAIDs in CVD have not been carried out but are needed (Table 2). Although the mechanism by which NSAIDs can cause mitochondrial dysfunction in cardiomyocytes is not fully understood, preventing increased mitochondrial ROS levels will reduce the adverse effects of elevated ROS levels and may reduce the incidences of CVD caused by NSAIDs.

Apart from the role of NSAIDs in causing cardiovascular incidences, NSAIDs affect other cardiovascular (CV) parameters including left ventricular function, infarct size, and blood pressure.In elderly patients (71.8 ± 7.6 years) NSAID exposure for <14 days showed a significantly higher left ventricular end-systolic dimension (+1.74 mm) and left ventricular end-diastolic dimension (+3.69 mm) with a significantly lower fractional shortening (−6.03%) when compared to non-NSAID users. Patients with NSAID use for >14 days were found to have higher left end-diastolic dimension (+1.96 mm) but no significant changes in other echocardiographic parameters compared to non-NSAID users [97].

The impact of NSAIDs on infarct size during myocardial infarction has only been investigated by a few laboratories. Indomethacin (10 mg/kg) was found to increase myocardial infarct size in animals, while ibuprofen (6.25 mg/kg/h) had the opposite effect [98–100]. The authors suggested that the opposite effect on the infarct size could be attributed to variable doses of the NSAIDs, different degrees of inhibition of prostaglandin and its by-products, and others factors like myocardial oxygen consumption [99]. The same group reported that the frequency of acute infarct expansion syndrome in patients with symptomatic pericarditis treated with indomethacin was 22% compared to ibuprofen which was only 8% [101]. The degree of infarct expansion was also greater in patients treated with indomethacin compared to ibuprofen [101].

Another important CV parameter is hypertension, which is one of the major contributors to the development of CVD. A series of events occur in patients with prolonged high blood pressure including left ventricular hypertrophy, systolic and diastolic dysfunction leading to arrhythmias, and heart failure [102]. Except for aspirin, all NSAIDs could potentially increase blood pressure when taken at doses necessary to alleviate pain and inflammation in both hypertensive and normotensive individuals [39].

NSAIDs through their ability to block COX enzymes lead to the inhibition of renal prostaglandin [103]. Renal prostaglandins modulate several renal functions which include maintaining renal homeostasis and exerting diuretic and natriuretic effects [104, 105]. Inhibition of the natriuretic effect of COX-2 leads to an increase in sodium retention thereby leading to excess water retention in humans [106]. The inhibition of renal vasodilating prostaglandins induces the production of vasoconstricting factors like vasopressin and endothelin-1. This also results in water retention, thereby leading to an increase in the total blood volume and causing altered systolic and diastolic blood pressure [103, 106, 107]. NSAIDs like ibuprofen, indomethacin, and naproxen increase the mean arterial pressure by 5 to 6 mmHg in patients with established hypertension [108, 109], which may be enough to raise medical concerns under certain conditions [110]. Additionally, the efficiency of all antihypertensive medications except for calcium channel blockers may be substantially reduced by NSAIDs [40]. Since CV outcomes would have different pathogenesis, increased ROS levels may not be the underlying mechanism for some occurrences of CVD. Other CV related parameters (such as hypertension), which are important for the normal functioning of the heart, are also likely to affect the CV outcome. Although direct comparisons between the different clinical studies shown in Table 2 are not possible, these studies suggest that CVD in NSAID users are influenced by the study populations used (such as with versus without preexisting acute myocardial infarction). Chronic use of NSAIDs by hypertensive individuals has been shown to increase the incidences of myocardial infarction, stroke, and cardiovascular mortality compared to nonchronic NSAID users [111] (Table 2).

## 7. NSAIDs and Nicotinamide Adenine Dinucleotide Phosphate-Oxidase

Apart from mitochondria being the major source of ROS generation, the plasma membrane bound nicotinamide adenine dinucleotide phosphate-oxidase (NADPH oxidase) has also been indicated in the production of ROS in phagocytes like neutrophils and macrophages where it produces an “oxidative burst” of O_2_^•−^ [112]. NADPH oxidases are considered to be the major source of nonmitochondrial ROS generation in many cells. The formation of free radicals is due to the electron transferred from the NADPH to molecular oxygen during the process of phagocytosis. The phagocytic NADPH oxidases play an important role in the host defense mechanism and are involved in killing pathogens ingested during phagocytosis. Apart from the phagocytic NADPH oxidase, in the last decade NADPH oxidase-dependent ROS generation was also identified in nonphagocytic cells including the endothelial cells, vascular smooth muscle cells, and cardiomyocytes of the cardiovascular system [113]. Compared to the phagocytic NADPH oxidases, the nonphagocytic enzymes produce lower amounts of ROS continuously and these levels may increase in the presence of specific extrinsic stimuli [113]. More interestingly, the ROS generated by NADPH oxidases lead to an enhancement in levels of ROS produced from other sources [112]. In one such study, mitochondria and NADPH oxidase 1 isoenzyme (Nox 1) were found to be closely coordinated for sustained generation of ROS leading to oxidative stress and cell death [112]. When human embryonic kidney 293T cells were exposed to serum-free media elevated ROS levels occur within 5 minutes of exposure and persisted for 8 hrs [112]. Utilizing RNA interference Nox isoenzymes were demonstrated to play a role in the induction of serum-withdrawal ROS generation. Although low levels of Nox 2 and Nox 4 did not affect the generation of serum-withdrawal induced ROS generation, low levels of Nox 1 led to a decrease in the ROS formation in these cells at 8 h (late phase). Using different mitochondrial complex inhibitors such as rotenone and potassium cyanide (KCN) mitochondria were found to play a role in the early phase (first 30 minutes) of ROS generation in the cells exposed to serum-free media [112]. The authors of the study suggested that mitochondrial generation of ROS occurs first, followed by the action of Nox 1 in the serum deprived cell system [112].

The adverse effect of NSAIDs on the cardiovascular system was evident by a study which reported that when male spontaneously hypertensive rats (SHR) were treated either with coxibs or with nonselective NSAIDs like diclofenac and naproxen, the mRNA expression of NOX enzymes 1, 2, and 4 was markedly increased in the heart and aorta [114]. Furthermore the oxidative stress in the heart and aorta of the NSAID treated animals was also elevated along with an increase in the O_2_^•−^ production. Of all the NSAIDs used, diclofenac was the most potent inducer of NADPH oxidases [114]. The role of NADPH oxidases in the generation of ROS in the animals was ascertained by studying the effect of apocynin (an NADPH oxidase inhibitor) in the reversal of the oxidative stress induced by diclofenac [114]. Activation of Nox 4 by NSAIDs like aspirin, naproxen, nimesulide, and piroxicam has also been reported in rat adipocytes resulting in higher production of H_2_O_2_ [115].

## 8. NSAIDs and Xanthine Oxidase

Xanthine oxidoreductase (XOR) is a conserved molybdoflavoenzyme occurring in milk and some tissues including the liver, heart, and small intestine [116]. It has two interconvertible forms: xanthine dehydrogenase (XDH) and xanthine oxidase (XO). Both enzymes catalyze the conversion of hypoxanthine to xanthine and xanthine to uric acid [116]. Although both enzymes are involved in purine degradation, XO is involved only in the reduction of oxygen while XDH is involved in the reduction of both oxygen and to a larger extent NAD^+^. The enzyme XDH has the ability to bind to NAD^+^ and, following the oxidation of hypoxanthine to xanthine, reduces NAD^+^ to NADH. On the other hand, XO, not being able to bind to NAD^+^, produces the free radical, O_2_^•−^ by the transfer of electrons to molecular oxygen. Apart from O_2_^•−^, H_2_O_2_ was also shown to be a major free radical generated by XO [117]. XDH can utilize molecular oxygen and generate ROS but to a lesser extent than XO. Compared to the other sources of ROS generation like mitochondria and NADPH oxidases, the role of XOR as a producer of ROS has been previously overlooked due to its relatively low activity in the heart of animals and human. Recently the role of XOR in mediating various pathological conditions has been demonstrated in the cardiovascular system due to significant increases in cellular XOR levels [118]. This was evident by the inhibition of XOR by compounds like allopurinol and oxypurinol which lead to a decrease in the oxidative tissue damage. Apart from their property of inhibiting XOR, allopurinol and oxypurinol have also been shown to act as free radical scavengers and inhibit lipid peroxidation as well as inducing heat shock proteins during times of oxidative stress [118]. Interestingly, a high level of serum uric acid, the end product of XOR, has been shown to be a marker for impaired oxidative metabolism [119].

Although the prevalence of this enzyme has been reported in human hearts [116, 120] and although increased levels of uric acid in myocardial heart failure have been reported in the literature [119], the role of NSAIDs on XO in the development of various cardiovascular conditions has not been investigated. In a study to investigate the adverse effect of aspirin on gastric mucosal lining leading to peptic ulcers and intestinal bleeding, rat gastric mucosal cells were incubated with aspirin and cytotoxicity already induced by hypoxanthine/XO was determined [121]. Aspirin was shown to increase the cytotoxicity induced by XO. This increase in cytotoxicity in the presence of hypoxanthine/XO was incorporated with an increase in uric acid levels which suggests ROS generation in these cells. Aspirin had an additive effect to these changes induced by hypoxanthine/XO [121]. In another study, it was seen that indomethacin augmented the XO activity in human colonic adenocarcinoma cells by more than 100% upon 60 minutes of exposure which resulted in a time dependent increase in the rate of lipid peroxidation [96].

## 9. NSAIDs and Lipoxygenase

Arachidonic acid that is formed by the action of diacylglycerol and cytosolic phospholipase A2 (cPLA_2_) from the membrane phospholipids is a substrate involved in the formation of either prostaglandins and thromboxanes or leukotrienes by the cyclooxygenase and lipoxygenase pathway respectively. The oxidation of arachidonic acid by these enzymes leads to the formation of ROS as the by-product [122]. It was shown that cPLA_2_-arachidonic acid linked cascade could lead to an increase in ROS generation via the lipoxygenase pathway in the rat fibroblasts [123]. The same group reported that the generation of ROS triggered by tumor necrosis factor- (TNF-) *α* was mediated by the activation of cPLA_2_ and the metabolism of arachidonic acid by 5-lipoxygenase [124]. Lipoxygenase has been reported to be involved in the upregulation of NADPH oxidases and increased ROS generation [125].

While NSAIDs are well known to inhibit the cyclooxygenase pathway, they do not inhibit the formation of leukotrienes by the lipoxygenase pathway. NSAIDs increases arachidonic acid levels [82, 126] and arachidonic acid itself can increase ROS generation [82]. Increased levels of leukotrienes and other metabolites of the lipoxygenase pathway induced by NSAIDs lead to gastric mucosal damage [127]. The effect of NSAIDs like indomethacin, piroxicam, and aspirin on the development of gastric lesions could be reversed by 5-lipoxygenase inhibitors and leukotriene antagonists [127]. This is consistent with NSAIDs increasing the levels of leukotrienes and products of 5-lipoxygenase activity. Increased occurrences of leukotriene C4 production were also observed in the gastric circulation induced by indomethacin [128]. The gastric and intestinal lesions in the presence of indomethacin could be reverted by 5-lipoxygenase inhibitor. Increased gastric mucosal leukotriene B4 production was observed in patients suffering from arthritis due to prolonged NSAID treatment [129]. NSAIDs have also been reported to increase the expression of 15-lipoxygenase-1 protein in colorectal cancer cells resulting in the inhibition of cell growth and apoptosis [130]. Although a growing body of evidence supports the idea that NSAIDs upregulate the lipoxygenase pathway, no direct evidence is available on the precise biochemical mechanism by which NSAIDs induced lipoxygenase pathway activity increase the rate of ROS generation. It is possible that the upregulation of the lipoxygenase pathway by NSAIDs may contribute to the net increase in free radicals leading to apoptosis and oxidative cell damage and ultimately cell death.

## 10. NSAIDs and Cytochrome P450

Cytochrome P450 (P450) is a family of biotransformation enzymes involved in critical metabolic processes [131]. They are a class of more than 50 enzymes that catalyze a diverse set of reactions with a wide range of chemically dissimilar substrates [131]. In humans they are primarily membrane-associated proteins that can be found in the inner membrane of the mitochondria or endoplasmic reticulum of cell [132]. They are predominantly expressed in the liver but are present in other tissues of the body as well.This group of proteins belongs to the heme-thiolate enzyme family, and they are involved in the oxidative metabolism of numerous compounds of endogenous and exogenous origin [133]. The role of P450 in drug metabolism has been considered to be a key factor in overcoming the adverse and toxicological effects of drugs [134]. The P450 system has been shown to play an important role in activation of oxygen and ROS generation [135, 136]. Normally, the active oxygen species are formed* in situ* during the P450 cycle when it reacts with a substrate. However, uncoupling of the P450 system results in excessive ROS generation and the P450 system being unable to metabolize a substrate, which leads to oxidative stress and subsequently cellular damage [133, 137].

Several studies have indicated the role of P450 in toxicity induced by NSAIDs [138–140]. One such NSAID, diclofenac, has been associated with hepatotoxicity due to poor metabolism by P450 [140]. Expression of a drug metabolizing mutant of P450, BM3 M11, in yeast mimicked the oxidative metabolite profiles of diclofenac metabolized by human P450s [141]. The treatment of the yeast cells expressing the mutant BM3 M11 with 30 *μ*M and 50 *μ*M diclofenac increased the ROS production in the cells significantly by 1.5 and 4 times, respectively, compared to cells not treated with the compound [140]. In another study by the same group, it was shown that diclofenac as well as indomethacin and ketoprofen showed an increase in ROS generation by 1.5–2 times compared to control [95]. On the other hand, P450 has been shown to be upregulated in bacterial cells by NSAIDs like ibuprofen, ketoprofen, and indomethacin by 11.8-fold, 3.9-fold, and 3.0-fold, respectively, relative to control cells [142]. Although no direct evidence is yet available on the involvement of P450 in the generation of ROS by NSAIDs in CVD, experimental data available in other cells suggest that NSAIDs may lead to increased ROS levels via the upregulation of P450 in cardiovascular related cells.

## 11. NSAIDs and Nitric Oxide Synthases

Nitric oxide (NO) is among the few gaseous biological messengers and can be synthesized from* l*-arginine by endothelial nitric oxide synthase (eNOS). NO has emerged as a key signaling molecule for maintaining vasodilation and the dysfunction of enzymes associated with NO production has been implicated in various pathological conditions including CVD, diabetes, and hypertension. When eNOS, which is responsible for regulating vascular homeostasis, is impaired due to a lack of the cofactor tetrahydrobiopterin (BH4), it results in the generation of O_2_^•−^ rather than NO [143]. This state is referred to as the uncoupled state of eNOS and is mainly attributed to the deficiency or lack of BH4 leading to the generation of free radicals [144]. The depletion of the BH4 can be the result of severe oxidative stress.

A 1.5-fold decrease in plasma nitrite levels was demonstrated in SHR treated with nonselective COX inhibitors and coxibs [114]. Although earlier reports demonstrated a cardioprotective effect of naproxen, a twofold decrease in plasma nitrite levels was observed after treatment of SHR with naproxen suggesting diminished levels of bioactive NO [114]. Additionally, the authors reported an upregulation in the eNOS mRNA expression levels rather than a decrease by diclofenac and naproxen by >2-fold. This increase has been attributed to the generation of H_2_O_2_ which increases eNOS expression at the transcriptional and posttranscriptional levels [145]. Diclofenac treated animals showed an increase in O_2_^•−^ production which could be inhibited by the NOS inhibitor* l*-NAME. NSAIDs have also been shown to impair the NO induced vasodilation in healthy individuals possibly due to increased oxidative stress induced by NSAIDs due to the uncoupling of eNOS [114].

## 12. NSAIDs, ROS, and Cardiovascular Diseases

Li et al. investigated the effect of several NSAIDs on free radical generation, NADPH oxidase expression, eNOS expression, and nitrite levels in the rat aorta and heart using a mouse model of hypertension as well as vasodilation in human subjects [114]. As previously described in the sections NSAIDs and nicotinamide adenine dinucleotide phosphate-oxidase and NSAIDs and nitric oxide synthases, this study demonstrated that the heart and aorta of the NSAID treated animals showed increased O_2_^•−^ production and oxidative stress. The oxidative stress was due to ROS formation by many enzymes including NADPH oxidases, with diclofenac being the most potent inducer of NADPH oxidases [114].

Rat embryonic H9c2 cardiac cells exposed to celecoxib, at concentrations of 10 *μ*M and 100 *μ*M, demonstrated a decrease in cell viability by 45% and 92%, respectively, and this was associated with a decrease in the expression levels of the cell survival protein Bcl2 [146]. However, 1 *μ*M of celecoxib had no significant effect on cell viability and was associated with an upregulation of the Bcl_2_ transcript level by ~30%. This effect of such a low amount of NSAID in the upregulation of cell survival gene expression is rather comparable to the effect of proteasome inhibitors like MG-132 or epoxomicin which at nanomolar ranges has been reported to actually increase the proteasome activity in the pretreated neocortical neurons [147]. Also, this was the first study that measured COX-2 levels directly in the cardiac cells [146]. Although no significant changes in the COX-2 levels were observed in cells treated with 1 *μ*M celecoxib, at 10 *μ*M the COX-2 levels decreased [146].

Apart from studying the effect of NSAIDs on cardiomyocytes, the role of NSAIDs on the COX enzyme system in platelets is also of prime importance. Platelets are important for the life-saving blood coagulation process [148] and are closely associated with CVD [149]. The aggregation of platelets by agonists like ADP, collagen, thrombin, or epinephrine leads to the formation of thrombus (microaggregate of platelets in fibrin mass), at the site of atherosclerotic plaque fissure on the coronary artery wall of the heart which results in thrombosis or CVD [150]. As expected because of the importance of platelets in CVD, the generation of ROS in platelets has been well-studied [149]. Measurement of O_2_^•−^ in platelets activated by thrombin as well as in inactivated platelets demonstrated that thrombin significantly increased the free radical (O_2_^•−^) generation by ~40% in the activated platelets compared to the inactivated platelets [149]. Although this result suggests the possibility of increased ROS formation in activated platelets, no direct role of NSAIDs in the production of ROS in the platelets has been reported [151]. However, it has been demonstrated that selective COX-2 inhibition led to an increase in platelet activation [152]. The increased platelet activation by COX-2 inhibition facilitated thrombotic vessel occlusion after the disruption of the vessel wall supporting a critical role for COX-2 inhibition in platelet activation and aggregation leading to thrombosis [152]. All these effects were accompanied by a decline in the production of endothelial prostacyclin. Diclofenac (1 mg/kg), a nonselective NSAID, caused significantly increased platelet vessel wall interaction with an increase in the number of adherent platelets on the endothelium of diclofenac treated animals compared to the control group [153]. Thrombotic vessel wall occlusion was also increased in animals treated with diclofenac once the vessel wall had been injured [153]. The direct contribution of ROS induced by NSAIDs to platelet activation and aggregation is yet to be determined. The role of aspirin in cardioprotection by the inhibition of platelet aggregation due to the inhibition of platelet thromboxane A2 is well established [154, 155]. Similarly, naproxen has also been suggested to have significant antiplatelet effect comparable to that of aspirin [156].

## 13. Possible Alternatives to NSAIDs for Reduced Cardiovascular Events

With the availability of a larger selection of synthetic drugs, people are more aware of the side effects of taking drugs on a regular basis. Nutraceuticals or phytoceuticals are a class of compounds that are derived from plants with fewer or no side effects compared to synthetic drugs [157] but with similar and sometimes even better beneficial biological effects [158]. Hyperforin obtained from the herb* Hypericum perforatum* has been reported to inhibit COX-1 3–18-fold higher when compared to aspirin [158]. Hyperforin is a natural antioxidant and has been shown to inhibit excessive ROS generation [159] as well as prostaglandin synthesis [160]. Several other studies indicate the usefulness of nutraceuticals in cardiovascular protection specifically by the inhibition of COX. Purified anthocyanins (pigment responsible for the color of fruits) have been shown to have an effect on COX activity [161]. While NSAIDs like naproxen (10 *μ*M) and ibuprofen (10 *μ*M) showed COX-1 inhibition of 47.3% and 54.3%, respectively, anthocyanins from raspberries (10 *μ*M) and blackberries (10 *μ*M) showed an inhibition of 45.8% and 38.5%, respectively. COX-2 inhibition by naproxen and ibuprofen was 39.8% and 41.3%, for sweet cherries it ranged from 36% to 48%, and for berries the inhibition was between 31% and 46% [161]. The antioxidant activities of the anthocyanins derived from these fruits were also high suggesting the importance of their role in reducing ROS.

In other studies the biologically active compounds of mycelia of* Grifola frondosa* and* Agrocybe aegerita* (edible mushrooms) were isolated and studied for their antioxidant and COX activity [162, 163]. The authors found that that fatty acids like ergosterol, ergostra-4,6,8(14),22-tetraen-3-one, and 1-oleoyl-2-linoleoyl-3-palmitoylglycerol in the case of* Grifola frondosa* and palmitic acid, ergosterol, 5,8-epidioxy-ergosta-6,22-dien-3beta-ol, mannitol, and trehalose in the case of* Agrocybe aegerita* as determined by spectroscopic analysis were the most potent in inhibiting both COX-1 and COX-2 [162, 163]. The efficiency of fish oil omega-3 fatty acids like eicosapentaenoic acid (EPA) and docosahexaenoic acid (DCHA) [164] in preventing CVD, stroke, and high blood pressure is also well established [165, 166]. It was suggested that fish oils have natural inhibitors which can inhibit COX [167]. While the EPA of fish oils inhibited COX-1 more than COX-2 [168], DCHA was shown to be an effective inhibitor of arachidonic acid induced prostaglandin biosynthesis [169]. Among the other reports available indicating the role of nutraceuticals in the cyclooxygenase pathway, calcitriol (vitamin D) [170], several types of flavonoids [171, 172], and marine derived steroids from formosan soft coral* Clavularia viridis *[173] all have been reported to affect prostaglandin synthesis. Interestingly most of the compounds exhibit antioxidant properties and inhibit both COX-1 and COX-2 [161, 172] suggesting a role in cardioprotection.

## 14. Conclusion

The review focuses on ROS generated by NSAIDs and its role in CVD. While the number of clinical experiments investigating the effects of NSAIDs on the cardiovascular events has significantly increased over the last two decades, basic research related to the mechanism by which NSAIDs cause cardiovascular dysfunction is limited. High variability in the clinical trials conducted (different populations, dosages, exposure, and types of NSAIDs) has led to results which are difficult to interpret and compare between studies. However, irrespective of the type of NSAID used, increased occurrence of CVD is common. Clinical trials showed that the risk of CVD is higher for coxibs than nonselective NSAIDs probably through the imbalance between prostacyclin/thromboxane levels.

According to the Joint Meeting of the Arthritis Advisory Committee and Drug Safety and Risk Management Advisory Committee, US FDA (2014), patients with a history of myocardial infarction, heart failure, hypertension, and other CV risk factors are at a greater risk of developing cardiovascular diseases due to NSAIDs usage compared to normal individuals [174]. The committee concluded that the increased risk of fatal cardiovascular thrombotic events, myocardial infarction, and stroke by NSAIDS should be lessened by using the lowest effective dose of NSAIDs for the shortest period of time possible.

Interestingly most of the studies that showed the generation of ROS induced by NSAIDs in various types of cells involved nonselective or semiselective NSAIDs (Table 3). However, irrespective of the type of NSAIDs used, either selective COX-2 inhibitors or the nonselective NSAIDs, NSAIDs produced oxidative stress with the nonselective NSAIDs showing a greater degree of ROS generation [114]. Therefore it may be hypothesized that it is through the generation of ROS as well as the upregulation and downregulation of several cell survival pathways that these NSAIDs exert their thrombogenic effect. Although COX-2 inhibitors have been shown to cause CVD some COX-2 inhibitors such as celebrex are currently still used widely, as the FDA determined the benefits of this drug outweigh the potential risks in properly selected and informed patients. The use of COX-2 inhibitors underscores the need for compounds with NSAID like properties without the side effects.

As such, various aspects of NSAID induced cardiotoxicity still need to be investigated, including the significance of NSAID induced lipoxygenase ROS generation in the cardiovascular system, determining if prevention of ROS production reduces CVD and determining if ROS production is needed for pain relief. Answers to these questions will result in substantial improvement on how CVD risk management will be conducted in the future.

## Figures and Tables

**Table 1:** List of NSAIDs with their recommended doses and levels in the circulation.

Generic name	Chemical name	Recommended dosages (may vary depending on the age and condition)	Levels in the circulation (*μ*M and *μ*g/mL) (dose dependent)	Trade name
Diclofenac	{2-[(2,6-Dichlorophenyl)amino]phenyl acetic acid	25 mg–150 mg/day	^*^5 *μ*M (1.5 *μ*g/mL) and 7 *μ*M (2.0 *μ*g/mL) [for 50 mg and 75 mg doses, respectively]	Zorvolex, Cataflam, Cambia, Voltaren, Voltaren gel, Arthrotec (combined with misoprostol), Flector, Zipsor, Pennsaid

Indomethacin	[1-(4-Chlorobenzoyl)-5-methoxy-2-methyl-1H-indol-3-yl]acetic acid	25 mg–200 mg/day	^*^3 *μ*M–6 *μ*M (1 to 2 *μ*g/mL)	Tivorbex, Indocin, Indocin SR, Indo-Lemmon, Indomethagan

Ketoprofen	2-(3-Benzoylphenyl) propanoic acid	25 mg–300 mg/day	^**^2 *μ*M to 22 *μ*M (0.43 to 5.62 *μ*g/mL) [50 mg dose]	Oruvail, Nexcede

Ibuprofen	2-(4-Isobutylphenyl) propanoic acid	100 mg–800 mg/day	^*^97 *μ*M–291 *μ*M (20–60 *μ*g/mL) [400 mg dose]	Advil, Motrin and Nurofen, Vicoprofen (combined with hydrocodone), Duexis (combined with famotidine)

Naproxen	(2S)-2-(6-Methoxy-2-naphthyl) propanoic acid	250 mg–1000 mg/day (both oral and IV)	^*^377 *μ*M (95 *μ*g/mL) [500 mg dose once every 12 h or 1000 mg dose for 5 days]	Naprosyn, EC-Naprosyn, Naprapac (copackaged with lansoprazole), *Aleve*, Accord, *Anaprox DS*, *Anaprox* Antalgin, Apranax, Treximet (combined with sumatriptan succinate) and Vimovo (combined with esomeprazole magnesium)

Sulindac	{(1Z)-5-Fluoro-2-methyl-1-[4-(methylsulfinyl)benzylidene]-1H-inden-3-yl}acetic acid	200 mg–400 mg/day	^**^1.4 *μ*M–6 *μ*M (0.5–2.0 *μ*g/mL) [constant for all doses]	Clinoril

Ketorolac	5-Benzoyl-2,3-dihydro-1H-pyrrolizine-1-carboxylic acid	10 mg–40 mg/day	^**^39 *μ*M (10 *μ*g/mL)	Toradol, Sprix

Piroxicam	4-Hydroxy-2-methyl-N-(2-pyridinyl)-2H-1,2-benzothiazine-3-carboxamide 1,1-dioxide	0.2 mg–20 mg/day	^*^5 *μ*M to 6 *μ*M (1.5 to 2 *μ*g/mL) [single 20 mg dose] 9 *μ*M to 24 *μ*M (3–8 *μ*g/mL) [for 20 mg repeated doses]	Feldene

Nimesulide	N-(4-Nitro-2-phenoxyphenyl)methanesulfonamide	100 mg twice daily	^**^10 *μ*M to 20 *μ*M (2.86 to 4.58) mg/L [100 mg] [175]	Sulide, Nimalox, Mesulid, Xilox, Nise, Nexen, Nidolon

Diflunisal	2′,4′-Difluoro-4-hydroxy-3-biphenylcarboxylic acid	250 mg–1000 mg	^**^164 *μ*M (41 *μ*g/mL) [250 mg doses] 348 *μ*M (87 *μ*g/mL) [500 mg doses] and 496 *μ*M (124 *μ*g/mL) [single 1000 mg dose]	Dolobid

Flurbiprofen	2-(2-Fluoro-4-biphenylyl)propanoic acid	50 mg–300 mg/day	^**^60 *μ*M (14 mg/L) [0.65 mg/kg of flurbiprofen IV] [176]	Ansaid

Mefenamic acid	2-[(2,3-Dimethylphenyl)amino]benzoic acid	250 mg followed by 500 mg every 6 hours	^*^83 *μ*M (20 *μ*g/mL) [250 mg single doses]	Ponstel

Tolmetin	[1-Methyl-5-(4-methylbenzoyl)-1H-pyrrol-2-yl]acetic acid	5 mg–1800 mg/day	^**^156 *μ*M (40 *μ*g/mL) [400 mg oral dose]	Tolectin, Tolectin DS, Tolectin 600

The table lists only those NSAIDs that have been mentioned in Table 3 along with their recommended doses and respective concentrations in the circulation. Most of the information regarding the dosages and levels in serum or plasma of the NSAIDs has been taken from the FDA approved site, http://www.drugs.com. Nimesulide is the only NSAID in the list that is not available in the USA. References for the plasma concentrations of flurbiprofen and nimesulide are given in the table. ^*^Serum concentrations, ^**^Plasma concentrations.

**Table 2:** Summary of results from clinical trials on the adverse effects of NSAIDs on cardiovascular diseases.

NSAID	Clinical trial name/location	Duration of study	Number of patients selected	Effect	References
Celecoxib, naproxen sodium, and placebo	Alzheimer's Disease Anti-Inflammatory Prevention Trial (ADAPT)/USA	4 years	2,528 patients with a family history of Alzheimer's Disease.	CVD/CBV death, MI, stroke, CHF, or TIA in the celecoxib-, naproxen-, and placebo-treated groups were 5.54%, 8.25%, and 5.68%, respectively. Death rates in the case of patients taking NSAIDs were higher but not statistically significant	[44]

Celecoxib, rofecoxib, valdecoxib, diclofenac, naproxen, ibuprofen, diflunisal, etodolac, fenoprofen, flurbiprofen, indomethacin, ketoprofen, ketoralac, meclofenamate, mefenamic acid, meloxicam, nabumetone, oxaprozin, piroxicam, sulindac, and tolmetin	None/USA	5 years	74,838 users of NSAIDs and 23,535 users of other drugs.	The adjusted rate ratio for Rofecoxib was 1.16 for MI and 1.15 for stroke which was the highest among all other NSAIDs including other coxibs. Naproxen modestly reduced the rate ratio (RR for MI 0.067 and RR for stroke 0.083) of cardiovascular events	[177]

Celecoxib, ibuprofen	None/Chieti, Chieti, Italy	3 months	24 patients undergoing aspirin treatment for cardioprotection	Ibuprofen interfered with the inhibition of platelet COX-1 necessary for cardioprotection by aspirin	[178]

Diclofenac, ibuprofen, indomethacin, ketoprofen, naproxen, mefenamic acid, piroxicam, tenoxicam, tolfenamic acid, aceclofenac, tiaprofenic acid, mefenamic acid, etodolac, nabumetone, nimesulide, and meloxicam, rofecoxib, celecoxib, valdecoxib, and etoricoxib	None/Finland	3 years	33,309 patients with first MI, 138,949 controls	Their results suggest that COX-selectivity do not determine the adverse effect of CVD by NSAIDs, at least concerning MI. No NSAID is MI-protective	[13]

Etoricoxib and diclofenac	Multinational Etoricoxib and Diclofenac Arthritis Long-term (MEDAL) Study Program/Multinational	18 months	34,701 patients (24,913 with osteoarthritis and 9,787 with rheumatoid arthritis)	Thrombotic cardiovascular events occurred in 320 patients in the etoricoxib group and 323 patients in the diclofenac group with event rates of 1.24 and 1.30 per 100 patient-years and a hazard ratio of 0.95 for etoricoxib compared to diclofenac. Long term usage of either of these NSAIDs had similar effects on the rates of thrombotic cardiovascular events	[179]

Etoricoxib and diclofenac	Etoricoxib versus Diclofenac Sodium Gastrointestinal Tolerability and Effectiveness (EDGE) trial/USA	9.3 months for etoricoxib and 8.9 months for diclofenac	7,111 patients etoricoxib (*n* = 3,593) or diclofenac sodium (*n* = 3,518)	The rate of thrombotic CV events was 1.30 and 1.24 in users of etoricoxib (90 mg) and diclofenac (150 mg), respectively, within 28 days	[180]

Ibuprofen, naproxen or lumiracoxib	The Therapeutic Arthritis Research and Gastrointestinal Event Trial (TARGET)/International	Extended report	18,325 patients with osteoarthritis	Ibuprofen increased risk of thrombosis and CHF compared to lumiracoxib (2.14% versus 0.25%) among aspirin users. Naproxen confers lower risk relative to lumiracoxib among nonaspirin users	[181]

Diclofenac, ibuprofen, and naproxen	None/USA	7 years	ND	Prolonged exposure to diclofenac increases the risk of AMI (relative ratio = 1.9–2.0) unlike ibuprofen or naproxen	[182]

Etoricoxib and diclofenac	EDGE II/USA	19.3 months for etoricoxib and 19.1 months for diclofenac	4,086 patients with rheumatoid arthritis etoricoxib *n* = 2,032; diclofenac *n* = 2,054	The rate of occurrence of AMI was higher in diclofenac (0.68) treated patients than in etoricoxib users (0.43). Also an overall increase in cardiac events was observed in diclofenac treated group (1.14) versus the etoricoxib group (0.83)	[183]

Celecoxib (at 400 mg QD, 200 mg two times a day (BID), or 400 mg BID)	None/USA	3 years	7,950 patients (with arthritis and other conditions)	The hazard ratio for the CVD, MI, HF, or thromboembolic event was lowest for the 400 mg once a day (1.1), intermediate for the 200 mg-BID dose (1.8), and highest for the 400 mg-BID dose (3.1)	[184]

Ibuprofen, naproxen, diclofenac, meloxicam, indomethacin, piroxicam, and mefenamic acid	None/UK primary care	20 years	729,294 NSAID users; 443,047 controls	Increase in the relative rate for MI with cumulative and daily dose of ibuprofen and diclofenac. Higher risk of MI with diclofenac use (relative ratio = 1.21) than ibuprofen (relative ratio = 1.04) or naproxen (relative ratio = 1.03)	[185]

Etoricoxib and diclofenac	The MEDAL Study/Multinational	19.4 to 20.8 months	Etoricoxib 60 mg/d; *n* = 6,769 90 mg/d; *n* = 5,012 Diclofenac *n* = 11,717	The thrombotic CV risk HR of etoricoxib to diclofenac = 0.96. Prolonged use of either NSAID resulted in an increased risk of thrombotic CV events	[186]

Naproxen, ibuprofen, diclofenac, celecoxib, and rofecoxib	None/USA	6 years	48,566 patients recently hospitalized for myocardial infarction, revascularization, or unstable angina pectoris	CVD risk increased with short term (<90 days) use for ibuprofen with incidence rate ratios of 1.67, diclofenac 1.86, celecoxib 1.37, and rofecoxib 1.46, but not for naproxen 0.88	[51]

Celecoxib, rofecoxib, valdecoxib, ibuprofen, naproxen, diclofenac, and indomethacin	None/USA	7 years	610,001 patients; without CVD *n* = 525,249; with history of CVD *n* = 84752	Rofecoxib (10.91 events/1000 person-years), valdecoxib (12.41 events/1000 person-years), and indomethacin (13.25 events/1000 person-years) increased CVD risk in patients with no history of CVD. Rofecoxib use increased risk of cardiovascular event in patients with CVD (30.28 events/1000 person-years)	[187]

Ibuprofen, diclofenac, rofecoxib, celecoxib, and naproxen	None/Danish population	9–34 days	153,465 healthy individuals	Dose dependent increase in cardiovascular events due to use of diclofenac (HR = 1.63), rofecoxib (HR = 2.13) and celecoxib (HR = 2.01) was observed	[188]

Celecoxib, ibuprofen, and naproxen	The Prospective Randomized Evaluation of Celecoxib Integrated Safety versus Ibuprofen Or Naproxen (PRECISION) trial/Multinational	2009-ongoing	20,000 patients with symptomatic osteoarthritis or rheumatoid arthritis at high risk for or with established CVD	This trial will determine the cardiovascular safety of the NSAIDs	[189]

ND	None/Denmark	10 years	107,092 patients with first incidence of HF	The hazard ratio for death due to MI or HF was 1.70, 1.75, 1.31, 2.08, 1.22, and 1.28 for rofecoxib, celecoxib, ibuprofen, diclofenac, naproxen, and other NSAIDs, respectively	[190]

Ibuprofen, diclofenac, naproxen, rofecoxib, celecoxib, valdecoxib, and etoricoxib	None/The Netherlands	4 years	485,059 subjects with first hospitalisation for acute myocardial infarction, CV, and gastrointestinal events	AMI risk with celecoxib (OR 2.53), rofecoxib (OR 1.60), ibuprofen (OR 1.56), and diclofenac (OR 1.51) was significantly increased. Significant increase in CV risk with current use of individual COX-2 inhibitors and tNSAIDs (OR from 1.17 to 1.64). Significant decrease in AMI with current use of naproxen (OR 0.48)	[191]

Ibuprofen, naproxen, diclofenac, etodolac, celecoxib, rofecoxib	None/Northern Denmark	10 years	32,602 patients with first atrial fibrillation or flutter and 325,918 population controls	Increased risk of atrial fibrillation or flutter was 40–70% (lowest for non-selective NSAIDs and highest for COX-2 inhibitors).	[192]

ND	None/Denmark	10 years	83,677 patients of which 42.3% received NSAIDs	Death/recurrent MI due to NSAID treatment (even short term) was significantly higher. Diclofenac posed the highest risk of all NSAIDs	[193]

ND	INternational VErapamil Trandolapril STudy (INVEST)/Multinational	7 years	882 chronic NSAID users and 21,694 nonchronic NSAID users. Patients with hypertension and clinically stable coronary artery disease	Primary outcome like all-cause mortality, nonfatal MI, or nonfatal stroke and secondary individual outcomes like all-cause mortality, cardiovascular mortality, total MI, and total stroke occurred at a rate of 4.4 events per 100 patient-years in the chronic NSAID group, versus 3.7 events per 100 patient-years in the nonchronic NSAID group	[111]

Celecoxib, rofecoxib, ibuprofen, diclofenac, naproxen, and others	None/Denmark	13 years (with first MI)	128,418 patients (77% participated in the study)	Incidences of coronary death or nonfatal recurrent MI with NSAIDs use remain unchanged with the time elapsed even after 5 years	[194]

ND	None/data obtained from REduction of Atherothrombosis for Continued Health (REACH) registry which includes patients from Latin America, North America, Europe, Asia, the Middle East, and Australia	4 years	4,420 NSAID users; 39,675 NSAID nonusers	1.16-fold higher risk of CVD, MI in NSAID users	[41]

MI: myocardial infarction, HF: heart failure, CVD: cardiovascular diseases, CBV: cerebrovascular diseases, CHF: congestive heart failure, HR: hazard ratio, OR: odd ratio, ND: not defined, RR: rate ratio, and TIA: transient ischemic attack. Various clinical trials suggest the increased incidences of CVD in NSAID users. The table lists only the clinical trials reported between 2006 and 2014.

**(a):** 

NSAIDs Nonselective/semiselective	Sources of ROS generation	Cells/models/animals studied	Outcomes	References
Diclofenac, indomethacin, ketoprofen	Mitochondrial respiration	Saccharomyces cerevisiae Yeast cells BY4741 and mitochondrial DNA deletion (rho^0^) strains	These NSAIDs target mitochondria to induce cell toxicity	[95]
Ibuprofen and naproxen			These NSAIDS induce toxicity independent of mitochondrial respiration	[95]

Indomethacin, sodium diclofenac, flurbiprofen, zaltoprofen, and mofezolac	ND	Human gastric epithelial cell line AGS	All NSAIDs except mofezolac increased apoptotic DNA fragmentation and expression of COX-2 mRNA. DNA fragmentation induced by indomethacin or flurbiprofen was reduced by antioxidants. Indomethacin at 1 mM was a potent inducer of ROS generation in the cells	[79]

Diclofenac and naproxen	NADPH oxidases	Spontaneous hypertensive rats	NADPH oxidase expression increased in the heart and aorta by diclofenac and naproxen. ROS levels increased due to NADPH oxidase	[114]
		Human EA.hy 926 Endothelial cells	Nox2 isoform of NADPH oxidase is increased by diclofenac	[114]

Aspirin, naproxen, and piroxicam	NADPH oxidases	Rat adipocytes	Activation of NOX 4 isoform of NADPH oxidase results in the generation of H_2_O_2_	[115]

Indomethacin	Xanthine oxidases	Human colonic adenocarcinoma cells (Caco-2 cells)	Xanthine oxidase activity increases by more than 100% 1 hour after treatment	[96]

Indomethacin	Mitochondria superoxide leakage	Rat gastric epithelial cell line RGM1	Indomethacin induced the leakage of superoxide anion in the isolated mitochondria from the gastric RGM1 cells	[93]

Indomethacin, diclofenac sodium, and aspirin	ND	Rat gastric epithelial cell line RGM1 and rat small intestinal epithelial cell line IEC6	Indomethacin, diclofenac, and aspirin in gastric RGM1 cells and small intestinal IEC6 cells increased lipid peroxidation	[93]

Sulindac, sulindac sulfide, and sulindac sulfone	ND	Pancreas carcinoma BXPC3, glioblastoma A172, colon carcinoma DLD-1, oral squamous SAS, and acute myelocytic leukemia HL60 cells	The NSAID sulindac and its metabolites sulindac sulfide and sulindac sulfone all increased ROS generation. Sulindac sulfide showed the greatest ROS generation	[80]

Indomethacin, piroxicam, and aspirin	Lipoxygenases	Gastric and intestinal mucosa in mice	Results in an overproduction of leukotrienes and products of 5-lipoxygenase activity. Increases in leukotriene and 5-lipoxygenase activity have been associated with ROS generation	[123, 124, 127]

Indomethacin	Lipoxygenases	Efferent gastric circulation of pigs	Time dependent formation of leukotriene-C4	[128]

Diclofenac	Cytochrome P450 enzymes	Saccharomyces cerevisiae yeast cells expressing the mutant P 450 BM3 M11 (capable of metabolizing diclofenac similar to humans)	Increased ROS production by ~1.5 and 1.8 times at concentrations of 30 *μ*M and 50 *μ*M, respectively, compared to wild type	[140]

Diclofenac, indomethacin, ketoprofen, and naproxen	Cytochrome P450 enzymes	Saccharomyces cerevisiae Yeast cells BY4741 and mitochondrial DNA deletion (rho^0^) strains	NSAIDs metabolism associated with increased P450-related toxicity	[95]

**(b):** 

NSAIDs coxibs	Sources of ROS generation	Cells/models/animals studied	Outcomes	References
Ibuprofen, ketoprofen, and indomethacin	Cytochrome P450 enzymes	*Bacillus megaterium *	All the NSAIDs caused a marked increase in the total cytochrome P450 level	[142]

Aspirin, diclofenac, diflunisal, flurbiprofen, ibuprofen, indomethacin, ketoprofen, mefenamic acid, naproxen, piroxicam, sulindac, and tolmetin	Cytochrome P450 enzymes	Rat hepatocytes	Cytotoxicity of diclofenac, ketoprofen, and piroxicam was increased by cytochrome P450 causing hepatotoxicity	[138]

Diclofenac and naproxen	Endothelial nitric oxide synthase	Spontaneous hypertensive rats	Increased expression of eNOS mRNA due to the generation of H_2_O_2_ which is responsible for upregulation of eNOS at the transcriptional and post-transcriptional levels	[114]

Rofecoxib and celecoxib	NADPH oxidases	Spontaneous hypertensive rats	NADPH expression increased in the heart and aorta by rofecoxib and celecoxib	[114]
		Human EA.hy 926 Endothelial cells	Nox2 Expression increased by rofecoxib	[114]

Rofecoxib and celecoxib	Endothelial nitric oxide synthase	Spontaneous hypertensive rats	In the aorta, the coxibs did not show any eNOS mRNA expression. In the heart only rofecoxib showed a significant increase in the expression of eNOS	[114]

Etodolac	ND	Human gastric epithelial cell line AGS	Increased apoptotic DNA fragmentation and expression of COX-2 mRNA	[79]

Nimesulide	NADPH oxidases	Rat adipocytes	Activation of NOX 4 isoform of NADPH oxidase results in the generation of H_2_O_2_	[115]

^*^Although other reports are available suggesting the role of NSAIDs in ROS formation, the table lists only those NSAIDs for which results show that these NSAIDs are associated with CVD or NSAIDs mentioned in the text.

## Abbreviations

CVD: Cardiovascular disease
COX-1: Cyclooxygenase-1
COX-2: Cyclooxygenase-2
NSAIDS: Nonsteroidal anti-inflammatory drugs
ROS: Reactive oxygen species.
